# Understanding Historical Demographic Processes to Inform Contemporary Conservation of an Arid Zone Specialist: The Yellow-Footed Rock-Wallaby

**DOI:** 10.3390/genes11020154

**Published:** 2020-01-31

**Authors:** Sally Potter, Linda E. Neaves, Mark Lethbridge, Mark D. B. Eldridge

**Affiliations:** 1 Division of Ecology and Evolution, Research School of Biology, Australian National University, Acton ACT 2601, Australia; 2 Australian Museum Research Institute, Australian Museum, 1 William Street, Sydney 2010, New South Wales, Australiamark.eldridge@austmus.gov.au (M.D.B.E.); 3 Royal Botanic Garden Edinburgh, 20A Inverleith Row, Edinburgh EH3 5 LR, UK; 4 Biological Sciences, Flinders University, Adelaide 5001, Australia; mark.lethbridge@flinders.edu.au

**Keywords:** marsupial, macropodid, conservation genetics, arid zone

## Abstract

Little genetic research has been undertaken on mammals across the vast expanse of the arid biome in Australia, despite continuing species decline and need for conservation management. Here, we evaluate the contemporary and historical genetic connectivity of the yellow-footed rock-wallaby, *Petrogale xanthopus xanthopus*, a threatened macropodid which inhabits rocky outcrops across the disconnected mountain range systems of the southern arid biome. We use 17 microsatellite loci together with mitochondrial control region data to determine the genetic diversity of populations and the evolutionary processes shaping contemporary population dynamics on which to base conservation recommendations. Our results indicate the highly fragmented populations have reduced diversity and limited contemporary gene flow, with most populations having been through population bottlenecks. Despite limited contemporary gene flow, the phylogeographic relationships of the mitochondrial control region indicate a lack of structure and suggests greater historical connectivity. This is an emerging outcome for mammals across this arid region. On the basis of our results, we recommend augmentation of populations of *P. x. xanthopus*, mixing populations from disjunct mountain range systems to reduce the chance of continued diversity loss and inbreeding depression, and therefore maximize the potential for populations to adapt and survive into the future.

## 1. Introduction

Habitat fragmentation, the division of natural habitat into small isolated patches, is associated with a myriad of problems for biodiversity worldwide. These include the interacting effects of small effective population size, isolation, contracted geographic distribution, novel species interactions, edge effects, and reduced landscape connectivity (see [1]). In particular, small isolated populations suffer loss of genetic diversity through random processes and a lack of immigration and increased inbreeding [2,3,4]. These genetic effects can impact the fitness of populations (inbreeding depression) and their ability to cope with change (environmental, climatic, disease), ultimately increasing extinction risk [4,5]. 

Fragmentation of natural landscapes has increased through anthropogenic impact, especially in the last 500 years, causing population and species decline and associated long-term genetic consequences which require active management to counteract [6]. While genetic data has been used to inform some conservation decisions (refer to [4]), the incorporation of such data is often limited in recovery plans for threatened species [7]. In natural landscapes, some species can avoid inbreeding through mate choice strategies and kin avoidance, population structure and sex-biased dispersal (e.g., [8,9,10,11,12]), but this is not always possible in fragmented landscapes. Therefore, understanding the genetic structure of populations, both contemporary and historical, is important to deciding how best to manage them, especially in fragmented landscapes, to ensure their long-term adaptability and survival [4,13]. This can be crucial for conservation programs where management requires captive breeding, translocations or reintroductions. We now recognize that the threat genetic erosion poses to small, isolated populations can be mitigated and often reversed through genetic management (genetic rescue), coupled with addressing threats (e.g., predator control) [4]. In most cases, genetic rescue involves supplementing inbred, low genetic diversity populations with individuals from another population to produce more genetically fit and viable populations for the future [4,6].

Australia has the greatest number of recent mammal extinctions in the world as a result of >200 years of European settlement [14]. Unlike other continents, the primary driver of declines and species extinctions is not human population pressures but rather the impact of introduced predators (e.g., European red fox *Vulpes vulpes* and the feral cat *Felis catus*), as well as changed fire regimes and habitat degradation by introduced herbivores [14,15]. This pattern of Australian mammal declines and extinctions continue [15,16,17,18] and additional research is required to understand the population dynamics of many declining species to assist in conservation planning and implementation. 

**Figure 1:**
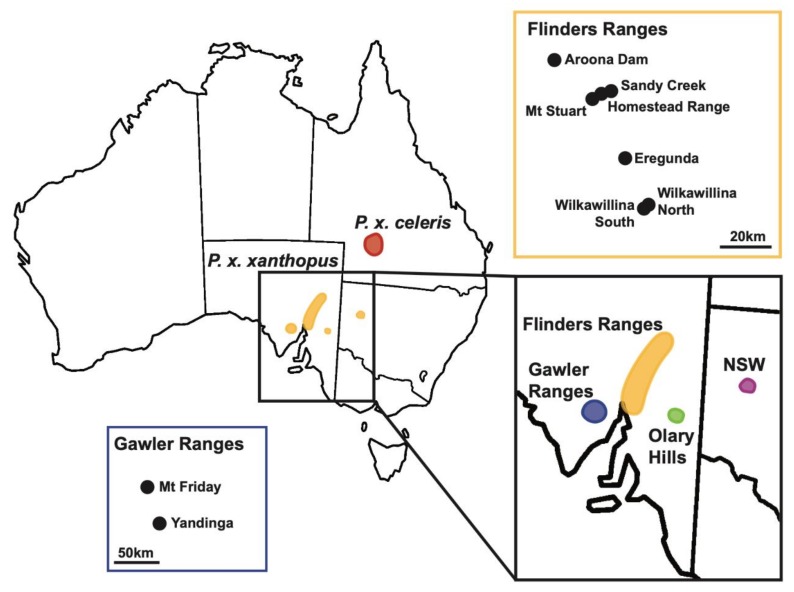
Map of the distribution of the yellow-footed rock-wallaby (*Petrogale xanthopus*) in Australia. The yellow populations outline the distribution of the subspecies *P. xanthopus xanthopus* in South Australia and New South Wales, and the red population highlights the distribution of *P. x. celeris* in Queensland. Inset, the populations and distributions of *P. x. xanthopus* from South Australia outline the distribution and populations across the Flinders Ranges (yellow), Gawler Ranges (blue), and Olary Hills (green) in South Australia, and the New South Wales (purple) population.

One species with a history of decline and the focus of ongoing management is the yellow-footed rock-wallaby (*Petrogale xanthopus*). The largest of the rock-wallabies, *P. xanthopus,* occurs in the semi-arid zone of southeastern Australia and consists of two subspecies, *P. x. celeris* in southwestern Queensland (QLD) and *P. x. xanthopus* in southeastern South Australia (SA) and western New South Wales (NSW) [19,20] (Figure 1), reported to have diverged ~140,000 to 590,000 years ago [21,22]. Only three of 17 rock-wallaby species (*P. xanthopus*, *P. lateralis*, and *P. rothschildi*) inhabit the semi-arid and arid zones of Australia, which occupies 70% of the continent [23]. Most *Petrogale* species occur in the more mesic eastern and northern Australia [20]. As their name suggests, rock-wallabies inhabit complex rocky habitat which naturally occurs patchily across the landscape. They rely on these rocky areas for dens, food, and protection, and occasionally disperse between habitat patches [20]. This habitat specificity and limited natural dispersal has important implications for the maintenance of genetic diversity within populations [24]. Currently, five *Petrogale* species are listed as ”threatened” [25](IUCN red list) and six species are ”near threatened”. Of these, four (including *P. xanthopus*) are now managed for conservation, as anthropogenic influences have fragmented their distributions and caused widespread population declines [26,27,28,29]. In several species, remnant populations have reduced genetic diversity because of small population size and reduced gene flow [30,31,32,33,34]. 

To date, genetic studies of *P. xanthopus* have been limited. Early research found genetic differentiation between *P. x. celeris* and *P. x. xanthopus* based on mitochondrial data [21,35]. In addition, studies within *P. x. celeris* revealed genetic connectivity among populations 10 km apart in connected habitat, but genetic differentiation between populations 70 km apart. Dispersal was limited between colonies [35]. There have been no population genetics studies yet for *P. x. xanthopus* throughout its range. 

Within *P. x. xanthopus,* there are major clusters of populations in SA, the Flinders Ranges, Gawler Ranges, and Olary Hills [19,36] and the Gap and Coturaundee Ranges in western NSW [37] (Figure 1). Once common, *P. x. xanthopus* has declined throughout its range, with many local population extinctions occurring in the last 150 years [19,29,36]. Declines have been attributed to habitat degradation from feral and domestic herbivores, as well as the impact of introduced predators [19,29]. The loss of habitat and populations, as well as the presence of exotic predators, means that the natural pattern of dispersal and gene flow among remaining YFRW populations is likely to have been disrupted across their distribution. In the past three decades, control of feral animals in some areas has resulted in the recovery of populations [29,38,39]. However, many surviving populations are small and population size is known to fluctuate with rainfall, with decline especially severe during drought [36,40]. *P. x. xanthopus* is listed as Vulnerable under the *Environment Protection and Biodiversity Conservation Act 1999* [41], with a total population size estimated to be 6500 adults [42].

Despite the increase in numbers in some populations, the ongoing effects of historic population extinctions and associated landscape changes means that a sound understanding of historic and contemporary genetic connectivity could aid future conservation efforts. Recent population viability analysis results predicting past and future responses to dispersal and population dynamics indicate high kinship coefficients [43]. Under three modeled translocation scenarios, there was no clear reduction of kinship coefficients over a 100-year time period, nor reconnection of two target area populations despite evidence of an initial connection at 50 years [43]. However, it is now understood that in the absence of ongoing natural gene flow, regular supplementations are required to counteract the impacts of genetic erosion and achieve genetic rescue [4,44].

The aims of this study were the following: (i) To assess genetic diversity and population structure of *P. x. xanthopus* across its range and (ii) assess contemporary and historical genetic differentiation and connectivity between populations and demographic processes shaping current patterns of diversity. We use a combination of mitochondrial sequences and microsatellite markers to inform conservation decision making for *P. x. xanthopus* and gain a better understanding of the evolutionary history of the arid zone of Australia. 

## 2. Methods

### 2.1. Study Area and Sampling

A total of 342 *P. x. xanthopus* were sampled from across their range in SA. Between 1998 and 2011, *P. x. xanthopus* were trapped as part of biannual monitoring by the Department of Environment and Natural Resources and Flinders University and ear biopsies collected. Eight sites in the Flinders Ranges, Eregunda (*n* = 65), Wilkawillina North (*n* = 26), Wilkawillina South (*n* = 75), Sandy Creek (*n* = 17), Mt Stuart (*n* = 3), and Homestead Range (*n* = 8); two sites in the Gawler Ranges, Yandinga Gorge (*n* = 91) and Mt Friday (*n* = 21); and one site in the Olary Hills (*n* = 11) (Figure 1 and Supplementary Table S1) were sampled. Within mountain ranges, sites were ~4 km to ~90 km apart, whilst the Flinders and Gawler Ranges are ~200 km apart, and Olary Hills and the Flinders Ranges are ~180 km apart (Figure 1). In addition, the reintroduced Aroona Dam population in the northern Flinders Ranges (*n* = 28) established from captive bred animals in 1996 [45] was sampled. For all populations, only adults or independent subadults were sampled, with pouch-young and young-at-foot removed from the analysis.

### 2.2. Molecular Analysis

DNA was extracted from ear biopsies using a salting out method [46]. 

#### 2.2.1. Mitochondrial DNA (mtDNA)

Mitochondrial sequence variation was determined by screening the hypervariable Domain I of the control region (*CR*) using marsupial-specific primers L15999M and H16498M [47]. PCR reactions were performed in 25 µL and included: ~100 ng genomic DNA, 10× PCR buffer (Qiagen, Hilden, Germany), 0.2 mM dNTPs, 2 mM MgCl_2_, 2 pmol primers, 5× Q-solution, and 0.5 U Taq DNA polymerase (Qiagen, Hilden, Germany). Thermocycling conditions were: denaturation at 94 °C for 2 min, 35 cycles of 20 s at 94 °C, 40 s at 55 °C, and 50 s at 72 °C, followed by a final extension for 5 min at 72 °C. PCR products were examined on a 1.5% agarose gel and then purified using USB^®^ ExoSAP-IT^®^ (Affymetrix, Cleveland, USA) and sequenced at the Australian Genome Research Facility (AGRF, Sydney, Australia) on an AB 3730*xl* DNA Analyzer (Applied Biosystems, Norwalk, USA). 

#### 2.2.2. Microsatellites

A total of 19 microsatellite loci were amplified in three multiplex PCR reactions (described in [48]). The primers used were derived from *P. x. celeris* (Y76, Y105, Y112, Y148, Y151, Y170, and Y175) [35,49], the allied rock-wallaby *P. assimilis* (Pa55, Pa297, Pa385, Pa593, Pa597, and Pa595) [50], the tammar wallaby *Notamacropus eugenii* (Me2, Me14, Me15, Me16, and Me17) [51] and the eastern grey kangaroo *Macropus giganteus* (G26.4) [52]. Samples were genotyped on an AB 3730*xl* DNA Analyzer (Applied Biosystems) at the AGRF (Melbourne, Australia). 

### 2.3. Data Analysis

#### 2.3.1. Genetic Diversity and Population Structure

Mitochondrial sequences were aligned and edited using MEGA v5.9 [53]. Previously published sequences from *P. x. celeris* (*n* = 7) and NSW *P. x. xanthopus* (*n* = 1) [35,54] were included for comparison. Diversity indices included: number of haplotypes (*H*); haplotype diversity (*h*), polymorphic sites, and nucleotide diversity (*π*) were estimated in DnaSP v5.10 [55]. 

Microsatellite genotypes were scored using GeneMapper v4.0 (Applied Biosystems). Conformance to Hardy–Weinberg equilibrium and linkage disequilibrium were tested by the Markov chain method in GENEPOP v.3.2 [56] using 1000 iterations, with the resultant *p*-values corrected for multiple tests using the sequential Bonferroni procedure [57]. Mean number of alleles per locus (*A*), as well as observed (*H_o_*) and expected heterozygosity (*H_e_*) were calculated using GenAlEx v6.5 [58,59], as well as the mean number of unique (uA) and rare alleles (*rA*) (allele frequency ≤ 0.05). Allelic richness (*AR*), the mean number of alleles per locus corrected for sample size (*n* = 8, Mt Stuart was excluded from this analysis as it had so few samples) was estimated using FSTAT v.2.9.1 [60], along with the proportion of inbreeding (*F*_IS_). The *F*_IS_ value for each population was tested using Weir and Cockerham’s estimator [61] with 1000 permutations. 

Population structure was estimated from the microsatellite data via two different approaches. First, a Bayesian clustering algorithm was run using the program STRUCTURE 2.3.1 [62] to estimate individual ancestry coefficients to determine distinct genetic populations. We used the admixture model, uncorrelated allele frequencies and lambda set to 1.0. Analysis was performed using all individuals and tested genetic clusters (*K*) ranging from 1 to 13. Each run included 10 replicates for each *K*, run for 1 million iterations after a burnin of 100,000 iterations. The inferred number of populations within the sample was deduced using both maximum posterior probability (L[*K*]; [62]) and maximum delta log likelihood (*∆K*) [63] implemented in CLUMPAK [64]. 

We also ran a principal coordinate analysis (PCoA) in GenAlEx to assess genetic structuring of the populations. This analysis used a covariance matrix with standardization based on genetic distances. This was conducted using the entire dataset, as well as smaller regional datasets (e.g., Flinders Ranges and Gawler Ranges) using a hierarchical approach to determine more fine-scale structuring.

#### 2.3.2. Relatedness Analyses

To assess the pattern of relatedness within and among populations we calculated the average pairwise relatedness in GenAlEx [59]. First, individual pairwise relatedness (r) was calculated using the Queller and Goodnight (1989) estimator [65]. Then, the average of pairwise values was calculated using the “Pops mean” analysis of the individual pairwise results. Significance was tested using 999 permutations and 1000 bootstrap resamplings to estimate 95% confidence intervals. Mean within population pairwise values were compared to upper and lower confidence limits estimated from a null hypothesis of no difference across populations. Relatedness (r) was assessed on the entire dataset and, then, separately for known male and female individuals to compare relatedness between the sexes.

#### 2.3.3. Population Differentiation

To assess relationships among mtDNA *CR* haplotypes, phylogenetic analysis was undertaken comparing the sequences from SA with published *CR* data from *P. x. xanthopus* in NSW [54] and *P. x. celeris* from QLD [35,54]. PartitionFinder v1.1.1 [66] was used to determine the best-fit model of DNA substitution for phylogenetic analysis based on the BIC, the raxml model and full search algorithm. This indicated the GTR + G model. Phylogenetic analysis was conducted using a maximum likelihood approach in RAxML v7.0.4 [67]. Analysis was conducted using the rapid bootstrap algorithm [68], with 100 bootstrap replicates and a random starting seed. Homologous sequences from a brush-tailed rock-wallaby (*P. penicillata*) and black-footed rock-wallaby (*P. lateralis*) were used as outgroups (GenBank accessions: HM136892.1 and AF348694.1, respectively). In addition, a haplotype network was estimated using TCS v1.21 [69] to visualize relationships of *CR* haplotypes, as low genetic variation can be challenging in resolving nodes in phylogenetic analyses. Sequence divergence among populations (Dxy) was also calculated using DnaSP. 

Pairwise differentiation of mtDNA haplotypes (*Φ_ST_*) among populations was estimated and tested for significance in Arlequin v3.5.1.2 [70] based on 110 permutations. For the microsatellite data, differentiation was assessed using pairwise *F*_ST_ calculated in Arlequin based on 110 permutations. An analysis of molecular variance (AMOVA) was conducted in Arlequin to examine the extent of population structuring within and among regional populations in SA (Flinders Ranges, Gawler Ranges, Olary Hills), significance was assessed using 1000 permutations. 

We tested for isolation-by-distance using a correlation approach following the methods of [71]. We compared matrices of genetic pairwise distance summed over all loci (microsatellite data) and log transformed pairwise geographic distance across populations using a paired Mantel test [72] in GenAlEx. Significant evidence for isolation-by-distance was assessed using 999 permutations on all individuals. Analysis was performed on the entire dataset, as well as on known males and females separately, to determine if there is sex-biased structure or gene flow.

#### 2.3.4. Demographic History Analyses

We tested for evidence of deviations from neutrality in mtDNA data using the Tajima’s D statistic [73] in DnaSP. We also estimated evidence of demographic expansion and selection/genetic hitchhiking using Fu’s Fs [74] and R2 tests [75] in DnaSP. In particular, the R2 statistic is powerful when dealing with limited sample size [75]. Tests were run on each population and significance of the estimates were assessed using 1000 coalescent simulations under a constant population size model.

To determine if populations showed any evidence of a recent genetic bottleneck, we ran the Wilcoxon’s heterozygosity excess test in the program Bottleneck v1.2 [76,77]. Data were examined using the two-phase model (TPM, [78]). 

#### 2.3.5. Migration Estimates and Connectivity

We estimated putative first-generation migrants between populations and their population of origin using the program Geneclass v2.0 [79]. This gives an estimation of contemporary dispersal between populations. We applied the Bayesian method [80] to estimate the likelihood that an individual originates from a given population and used the Monte Carlo resampling method following the method of [80]. We applied the statistical criterion, L_home/L_max, the ratio of L_home—the likelihood of the individual within the population it is sampled to the highest likelihood value among all sampled populations including the population where the individual was sampled (L_max). The L_home/L_max ratio has more power than the L_home statistics (see [81]). Individuals that were significantly different from their sampled population were only assigned to another population if the significance was P < 0.01. Immigrants that were still significant *P* < 0.05, were considered immigrants from unsampled populations. Detected migrants were checked for concordance with the STRUCTURE results.

## 3. Results

### 3.1. Genetic Diversity and Population Structure

#### 3.1.1. MtDNA

A total of 28 *CR* haplotypes (696 base pairs (bp) and 644 bp when outgroup indels removed) were identified across *P. x. celeris* (seven haplotypes) and *P. x. xanthopus* (21 haplotypes), see Supplementary Table S1 for individual haplotypes and GenBank accessions (MN781209-MN781237). For *P. x. xanthopus*, this comprised 14 haplotypes in the Flinders Ranges, three in the Olary Hills, three in the Gawler Ranges, and one from NSW. There was a total of 42 polymorphic sites and three indels among *P. xanthopus* haplotypes. Overall haplotype diversity within *P. x. xanthopus* was 0.843 and nucleotide diversity was 0.013. Haplotypic diversity ranged from 0.000 to 1.000 and nucleotide diversity ranged from 0.000 to 0.0218 (Table 1). No haplotypes were shared between regions, but three haplotypes were shared between sites (haplotypes Bt and Bc between Wilkawillina North and South, and haplotype H between Mt Stuart, Homestead Range, and Sandy Creek, Table 1). 

#### 3.1.2. Microsatellites

Locus Pa55 was monomorphic in most (six) populations and so was removed from further analyses. Four other loci were monomorphic in some populations, Pa385 and Y105 in Mt Stuart, Y175 in Mt Friday, and Y148 in Aroona Dam. Remaining loci were polymorphic across all populations. All loci in all populations were in HWE (*P* > 0.05), except for Pa593 (Sandy Creek), Y151 (Sandy Creek), Me15 (Eregunda), Y112 (Wilkawillina South), G26-4 (Wilkawillina North), and Pa595 (Aroona Dam, Wilkawillina North, Sandy Creek) after sequential Bonferroni corrections. However, there was no consistent pattern in loci across populations, except for Pa595 which was removed from further analyses. Sandy Creek was out of HWE at four loci; however, we kept this population in the analysis. There was no consistent evidence of LD with only 1.3% of pairwise comparisons significant after sequential Bonferroni correction. 

A total of 157 alleles were identified across populations, including 92.4% (145 alleles) in the Flinders Ranges and 44.6% (70 alleles) in the Gawler Ranges. The mean number of alleles per locus (A) ranged from 2.7 in Yandinga to 6.1 in Wilkawillina South (Table 2). Unique alleles were detected in all populations and ranged from 6% in Mt Stuart and Wilkawillina North to 41% in Mt Friday (Table 2). Rare alleles varied from 0% (Homestead Range and Mt Stuart) to 19.7% (31/157 alleles, Wilkawillina South) and included a total of 106 alleles in the Flinders Ranges and 15 alleles in the Gawler Ranges. Allelic richness which was adjusted for variation in sample size (excluding Mt Stuart), ranged from 2.23 in Yandinga to 4.34 in Wilkawillina South (Table 2). Observed heterozygosity (Ho) ranged from 0.38 to 0.73 (Yandinga, Homestead Range) and He ranged from 0.37 to 0.66 (Yandinga, Wilkawillina South) (Table 2). No significant inbreeding (F_IS_) was detected for populations, with values ranging from −0.234 in Mt Stuart to −0.027 in Wilkawillina South (Table 2). 

#### 3.1.3. Population Structure

**Figure 2:**
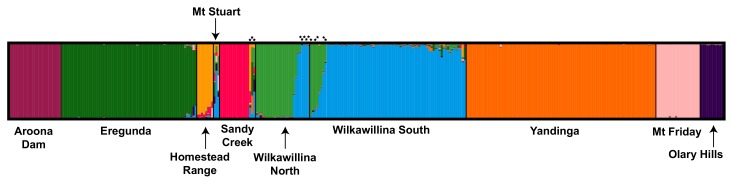
STRUCTURE plot outlining the proportional membership (Q) of each *P. x. xanthopus* individual (represented by a single vertical bar) into genetic clusters. The populations are labelled and highlighted by black lines. Star (*) represents first generation migrants detected in GeneClass2 analyses (see Supplementary Tables S5 and S6 for details).

The Bayesian model-based clustering analysis implemented in STRUCTURE indicated that either nine (maximum *L(K)*) or two (maximum Δ*K*) populations were present in the 10 sampled sites. At *K* = 9, CLUMPAK indicated a major cluster (9/10) supporting separation of all sites, excluding Mt Stuart which was shown as highly admixed, perhaps as a result of its small sample size (*n* = 3). Admixture was evident between Wilkawillina North and South, as well as between Sandy Creek and multiple other Flinders Ranges populations (Figure 2). The minor cluster (1/10) had similar structure, however, the Homestead Range revealed a greater level of admixture with Sandy Creek. At *K* = 2, Yandinga Gorge was separated from all other populations (results not shown). On the basis of the *K* = 2 conundrum (see [82], where a higher proportion of *K* = 2 clusters were identified using the *∆K* than other methods, we present the highest probability results only. 

The PCoA identified Yandinga Gorge (Gawler Ranges) as the most differentiated population, with Aroona Dam (reintroduced Flinders Ranges) and Mt Friday (Gawler Ranges) also forming a distinct grouping (Supplementary Figure S1a). PC1 accounted for 21.8%, and PC2 for 7.9% of the variation. When analysed by region (e.g., Gawler Ranges vs. Flinders Ranges), the PCoA results indicate strong differentiation of the two sampled Gawler Ranges populations (Yandinga Gorge and Mt Friday, Supplementary Figure S1b). These populations differentiate on PC1 which accounts for most of the genetic variation (PC1 = 36.6%, PC2 = 7.1%). For the Flinders Ranges, Aroona Dam was the most differentiated on PC1 (12.1%) whilst PC2 (8.8%) separated Eregunda and Sandy Creek from the remaining North and South Wilkawillina populations and Mt Stuart which were all clustered. The Homestead Range clustered slightly separately from this main cluster on PC1 (Supplementary Figure S1b).

### 3.2. Relatedness

**Figure 3:**
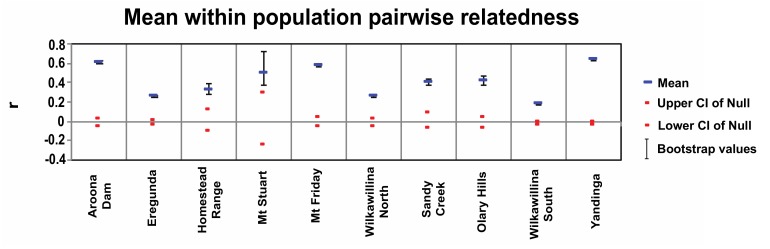
Within population mean pairwise relatedness (r) for *P. x. xanthopus* from 10 populations in South Australia. Calculations followed the method of [65] with the 95% upper and lower confidence bounds around the null hypothesis of no difference across populations (red). Error bars surround the mean pairwise relatedness (r, blue line) based on 1000 bootstrap resampling.

Mean pairwise relatedness was significant in all populations (*P* < 0.02), ranging from 0.185 in Wilkawillina North to 0.644 in Yandinga (Figure 3). All relatedness values fell above the 95% confidence limits based on the permutations of a null hypothesis of ”no difference”, indicating individuals are more related than by chance. This is most evident for Aroona Dam, Mt Friday, and Yandinga. When males and females were analyzed separately, the mean relatedness was significant in all populations (*P* < 0.03), with r values being more related than expected by chance. However, the mean fell within the null hypothesis bounds for Homestead Range for both males and females, and Sandy Creek for males (Supplementary Figure S2).

### 3.3. Population Differentiation

**Figure 4:**
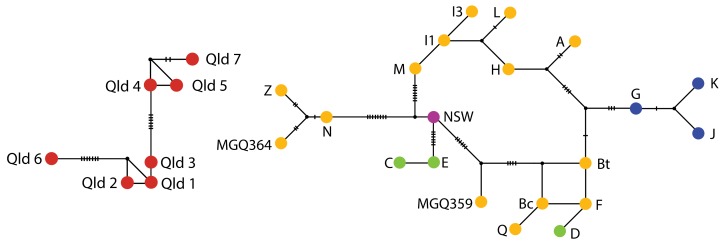
Haplotype networks based on analysis of haplotypes from the mitochondrial control region of *P. x. xanthopus* and *P. x. celeris* (red). The red Qld samples were too distantly related to the *P. x. xanthopus* haplotypes to form a single network. Haplotypes from *P. x. xanthopus* are outlined in Supplementary Table S1 and the unresolved phylogenetic tree in Supplementary Figure S3. Yellow samples correspond to populations from the Flinders Ranges, blue from the Gawler Ranges, green from Olary Hills, and purple from NSW (refer to Figure 1 for map).

The phylogenetic relationship among mtDNA *CR* haplotypes supported the distinction of *P. x. celeris* from *P. x. xanthopus*. The relationship within *P. x. xanthopus* haplotypes, however, did not support any further geographic structuring (haplotype network, Figure 4). Although there was some geographic clustering of related *P. x. xanthopus* haplotypes, these are all part of one monophyletic clade with low internal branch support, indicating historic connectivity across populations (Supplementary Figure S3). Sequence divergence between populations ranged from 0% to 2.5% based on Dxy (Supplementary Table S2). 

Significant population differentiation was detected between all populations for mtDNA (*Φ_ST_*) except between Homestead Range and Mt Stuart. Values ranged from 0.00 to 1.00 (Table 3). Significant population differentiation was detected between all populations based on the microsatellite data (*F*_ST_) with values ranging from 0.050 between Wilkawillina North and Wilkawillina South in the Flinders Ranges to 0.498 between Yandinga and the reintroduced Aroona Dam population (Table 3).

The AMOVA results indicated the majority of variation (61.6%) is between populations within groups, with only 28.2% variation among Gawler Ranges, Flinders Ranges, and Olary Hills. Within populations, there was 10.2% variation which was not significant as compared with the variation within and among populations and groups.

Significant correlations of genetic divergence and log transformed geographic distance (P(random Rxy) ≥ Rxy from the data, *P* = 0.001) were detected for *P. x. xanthopus*, indicating isolation-by-distance among populations. The Mantel test estimated a moderate correlation (Rxy = 0.666) based on the entire dataset. When males and females were analyzed separately, the correlations were significant (*P* = 0.001), with much higher correlations for females (Rxy = 0.812) than for males (Rxy = 0.672, see Supplementary Figure S4).

### 3.4. Demographic History

Tests of neutrality (Tajima’s D) and expansion (Fu’s Fs and R2) provided little evidence for selection or expansion across populations based on the *CR* sequence data (see Supplementary Table S3). Simulations of Fu’s Fs and R2 under a constant population model were nonsignificant for all populations, despite significant estimates of Fs for Wilkawillina South (*P* = 0.009) and Olary Hills (*P* = 0.049). Tajima’s D results were significant for Sandy Creek and Olary Hills suggesting evidence of deviations from neutrality (*P* < 0.05 and *P* < 0.01, respectively) and simulations were significant for these two populations and Eregunda under a constant population size model. Tests of neutrality were unable to be computed for Aroona Dam, Yandinga, and Homestead Range due to a lack of polymorphisms in these populations and Mt Stuart due to the small sample size.

The Wilcoxon’s heterozygosity excess test revealed multiple populations had significant deviations from drift/mutation equilibrium, evidence of recent genetic bottlenecks. Genetic bottlenecks were detected for Aroona Dam, Eregunda, Homestead Range, Mt Friday, Wilkawillina North, Wilkawillina South, and Yandinga based on the TPM model (Supplementary Table S4). However, results for most of these populations (excluding Eregunda, Wilkawillina South, and Yandinga) should be interpreted with caution as their sample size is less than 30 individuals, which is the recommended sampling for this analysis [76].

### 3.5. Migration and Connectivity

A total of 13 putative first-generation migrants were detected in three populations: Wilkawillina South, Wilkawillina North, and Sandy Creek (Supplementary Table S5). Five individuals from Wilkawillina North were predicted to have come from Wilkawillina South and likewise five individuals from Wilkawillina South were predicted to have migrated from Wilkawillina North (2.4 km apart). In addition, three individuals from Sandy Creek were detected as migrants from Homestead Range, Wilkawillina North, and Wilkawillina South (~3 to 60 km apart, refer to Table 3).

## 4. Discussion

Genetic analysis of *P. x. xanthopus* populations across southeastern Australia has found strong fine-scale contemporary structuring both within and between mountain range systems (Figure 2). However, indications of greater historical connectivity suggest that recent fragmentation has increased contemporary population structure. Evidence of isolation-by-distance among populations and limited contemporary gene flow is present, even within extensive mountain range systems with widespread historically suitable habitat. Therefore, it appears the recent recovery of some populations has not resulted in widespread gene flow. Our results support growing evidence that fragmentation and reduced habitat suitability increases isolation among remnant populations, causing a decline in species persistence, abundance, richness, and ecosystem dynamics [83]. This is consistent with previous population viability analyses that revealed high kinship coefficients among a subset of these populations [43]. The low genetic diversity, greater relatedness than by chance, and evidence of bottlenecks within populations indicates that conservation management initiatives are needed to change the trajectory of many *P. x. xanthopus* populations.

### 4.1. Contemporary Population Structure and Genetic Diversity

Microsatellite and mtDNA analyses found strong fine-scale contemporary genetic structuring both within and between mountain range systems. Genetic clustering of microsatellite data indicates the presence of nine distinct groupings that largely comprise the sampled populations. Populations within the Gawler Ranges were distinct, however, the PCoA results indicate some overlap of Olary Hills with populations from the Flinders Ranges (Supplementary Figure S1a) and some evidence of admixture in the Flinders Ranges (Figure 2). Despite this, sampled populations were all significantly divergent based on *F*_ST_ (0.050 to 0.498, Table 2). The *F*_ST_ values are similar or greater than those reported from other rock-wallaby populations which inhabit heavily modified landscapes using similar microsatellite loci, including *P. penicillata* (0.072, <10 km apart) [84] and *P. lateralis* (0.238, <10 km apart) [33]. They are also similar to *F*_ST_ values between populations for *P. x. celeris* from QLD at equivalent distances (0.238, 10–70 km) [35]. They are, however, much higher than the values estimated in unmodified landscapes, for example, *P. brachyotis*, *F*_ST_ = 0.027–0.059, <67 km apart [85] and *P. wilkinsi*, *F_ST_* = 0.085, 1.2 km apart [86]. The AMOVA results further support strong population structure at a fine scale, with the greatest structure between populations (61.6%), then, between regions (e.g., Flinders Ranges, Gawler Ranges, and Olary Hills 28.2%). These high levels of differentiation indicate limited contemporary gene flow between populations within the Flinders Ranges, Gawler Ranges, and Olary Hills, as well as between these regions. The larger *F*_ST_ values between more distant populations is in accordance with the isolation-by-distance results which support greater genetic differentiation with distance. Such isolation-by-distance results, particularly among females, are a common finding among rock-wallaby species [12,85]. 

Contemporary differentiation was similarly detected between most populations from the mitochondrial *CR* (*Φ_ST_*) (Table 2: excluding Homestead Range and Sandy Creek). However, there was little evidence of phylogeographic structure among the major range systems (Figure 4) (discussed below). Average genetic differentiation (*Φ_ST_*) within range systems was 0.75 to 0.89 (Flinders Ranges and Gawler Ranges, respectively) as compared with average genetic differentiation between range systems (0.88 to 0.95, Flinders Ranges and Olary Hills vs. Gawler Ranges and Olary Hills). The lowest levels of differentiation are between geographically closer populations (e.g., Wilkawillina North and Wilkawillina South) but overall maternal gene flow is limited. Strong female philopatry has been reported in other *Petrogale* (*P. penicillata*, [12,87,88]; *P. brachyotis*, [85]) and is a common feature of mammalian systems, together with male-biased dispersal [89,90,91]. This is also consistent with previous findings of average dispersal at 2 km for females and 4.5 km for males in *P. xanthopus* from population viability analysis [43]. 

Despite strong population structure, a few sampled populations in the Flinders Ranges, including the geographically proximate Wilkawillina North and Wilkawillina South (4 km apart) did show evidence of recent connectivity (shared mtDNA haplotypes and admixture of microsatellite genotypes), with first generation migrants detected between these sites. Shared *CR* haplotypes were found between Homestead Range, Mt Stuart, and Sandy Creek within the Flinders Ranges (Table 1) and evidence of first-generation migrants between Sandy Creek and Homestead Range, Wilkawillina North and Wilkawillina South (Supplementary Tables S5 and S6 ), suggest the Flinders Ranges populations may be better connected. Eight additional individuals showed evidence of admixture from STRUCTURE results (>0.70) but were not detected as first-generation migrants and could represent backcrosses (Figure 2 and Supplementary Table S5). The mixed ancestry detected for Mt Stuart could be a consequence of the small sample size (*n* = 3) for this population and associated lack of genetic information to form a distinct cluster (Figure 2). Interestingly, most of the individuals that showed admixture are males and carry *CR* haplotypes that differ from those typically found in the population they were trapped in (Supplementary Tables S5 and S6). Some of these individuals were only detected once in the population, so it is difficult to detect if these individuals were successful in mating, contributing to subsequent genetic diversity. They could represent floating individuals or individuals that did not survive or reproduce after dispersal. Further research is required to determine if migrants contributed to the genetic diversity in their new populations through parentage analyses. Not all populations were sampled. Further analysis incorporating additional populations which are located both between currently sampled populations and outside (e.g., in Flinders and Olary Ranges) could identify additional immigrants and first-generation migrants. 

The microsatellite diversity, although similar to other remnant populations of rock-wallabies (e.g., [92]), was still rather low (allelic richness 2.23 to 4.34, Table 2). Despite extensive sampling for some populations (e.g., *n* = 91, Table 2), allelic diversity and richness was low, in general, across range systems (average allelic diversity 2.89 to 4.16 and allelic richness 2.5 to 3.75, for Flinders Ranges and Gawler Ranges, respectively). Most population genetic studies to date have focused on threatened and highly fragmented populations (e.g., *P. penicillata* and *P. lateralis* [30,32,33,84,88]) and as such, the comparisons are biased to already disrupted populations. These low levels of diversity are likely a consequence of population decline and recovery after a bottleneck or captivity. Bottlenecks were detected in up to seven populations, including Aroona Dam, a population developed from captive bred stocks, Yandinga and Mt Friday which all show low heterozygosity (0.38 to 0.52) and allelic richness (2.23 to 2.77, Table 2). Despite low diversity within populations, our results indicate no significant evidence of inbreeding within any of the populations (F_IS_, Table 2). Each population contained unique alleles and rare alleles were present in 80% of the populations (Table 2). Other rock-wallaby species (e.g., *P. penicillata*) have been shown to mitigate inbreeding through mate choice and sex-biased dispersal, with female philopatry and male-biased dispersal documented to assist in inbreeding avoidance [12,88,93]. 

The mtDNA diversity within populations was extremely low and nucleotide and haplotype diversity was zero for four populations (Aroona Dam, Homestead Range, Mt Stuart, and Yandinga). The haplotype numbers varied from one to six, with most populations only having one or two distinct *CR* haplotypes, despite up to 100 individuals being sampled. Evidence of bottlenecks across populations (Supplementary Table S4) may have influenced this lack of mtDNA diversity but it could also be a product of limited female dispersal. 

### 4.2. Phylogeography and Historical Connectivity

Although population genetic analyses of the mitochondrial DNA data (*Φ_ST_*) indicate significant contemporary differentiation between most populations (Table 1, excluding Homestead Range and Sandy Creek), there is little evidence of phylogeographic structure among the major range systems (Figure 4). Only haplotypes from the Gawler Ranges formed a cluster of related haplotypes (Figure 4). However, Mt Friday and Yandinga did not form a strongly supported monophyletic lineage under phylogenetic analysis (Supplementary Figure S3). The low sequence divergence reported across this highly variable mitochondrial region (0% to 2.5%), shared haplotypes between some populations and limited geographic structuring of haplotypes suggests historic connectivity among populations right across the region, even between the NSW and SA population isolates (Figure 4 and Supplementary Table S2). This is similar to findings reported in the congener *P. lateralis*, from the central Australian arid zone and revealed historical connectivity across disjunct populations >300 km apart [34]. This similarity suggests a common impact of climatic history in shaping patterns of diversity in these species. 

Despite the arid biome forming during the Miocene as a result of Australia’s movement northward, many species inhabiting this region show only recent divergence [23]. One hypothesis is that range systems acted as refugia across the landscape during Pleistocene climatic changes (see [23]). However, previously we did not know whether the range systems harbor a large proportion of diversity, nor if these ranges were connected. Our results emphasize the deep divergence between the subspecies *P. x. celeris* and *P. x. xanthopus* (Figure 4 and Supplementary Figure S3) but highlight that the Gawler and Flinders Ranges, Olary Hills, and ranges in western NSW were historically linked by gene flow, indicating broad connectivity and persistence across the region. The AMOVA results revealed a stronger population structure within range systems than between ranges across the landscape. This supports our notion of greater historical connectivity between the Flinders Ranges, Gawler Ranges and Olary Hills.

While single species studies are important for addressing existing in situ conservation concerns, a broader understanding of the historical evolutionary processes shaping genetic patterns of diversity is also valuable. Although our knowledge of Australian biome history is improving, particularly for the mesic east and south (e.g., [94,95,96,97]) and more recently the monsoonal tropics [98], we still lack a deep understanding of the biodiversity structure and history across the arid and semi-arid zones (i.e., arid biome) [23]. There is little phylogeographic or population data for this region, which encompasses more than half the continent and includes numerous deserts and range systems. Results to date indicate varied faunal responses to past climatic changes, including isolation of populations in multiple refugia [99,100,101,102,103,104,105,106], as well as transcontinental connectivity for some species (e.g., [107,108,109,110,111,112]). Few studies have examined mammals, and this is only the third comprehensive analysis of a mammal species from the Australian arid biome [34,113]. The research on congener *P. lateralis* and the sandhill dunnart (*Sminthopsis psammophila*) both revealed similarities in historical connectivity across the landscape, highlighting the need to maintain genetic diversity across the landscape to enable resilience of species across this heterogeneous environment. Comparison and analysis across diverse organisms would assist in identifying core refugial areas where species have persisted across the landscape through past climatic cycles and improve our knowledge of biodiversity hotspots, as well as highlight areas of importance for conservation to establish evolutionary resilience (e.g., [114,115].

### 4.3. Implications for Conservation Management of P. x. xanthopus

Our results highlight the need for conservation management of *P. x. xanthopus*. If the currently limited dispersal and gene flow continues, it will have long-term negative consequences for genetic diversity and survival of populations. Recent genomic evidence from the helmeted honeyeater (*Lichenostomus melanops cassidix*) demonstrates how individuals with weak signatures of inbreeding depression can have fitness declines and strong lifetime effects in reproductive success [116]. Despite no current evidence of inbreeding, the low genetic diversity, high average relatedness, and evidence of past bottlenecks suggests management action needs to be considered, to increase genetic diversity within populations and create longer term stability and connectivity among populations. The two populations in the Gawler Ranges have low diversity, show evidence of recent bottlenecks, and no signs of current gene flow. Genetic differentiation between Yandinga and Mt Friday are similar to differentiation comparisons with populations from the Flinders Ranges, ~200 km away and are likely the result of drift in small populations (e.g., [117]). Likewise, the reintroduced Aroona Dam population in the Flinders Ranges, founded from a captive colony, also displays low genetic diversity. Some of our diversity and admixture results should be taken with caution as population sampling is low. However, Mt Stuart (*n* = 3) for example, the sample represents the entire extant remnant population at the time. Overall, our results highlight the need for focused and broad action for genetic management of *P. x. xanthopus*, to improve genetic diversity and rebuild connectivity, at least within mountain range systems. Furthermore, where possible in the future, additional genetic sampling coud aid in conservation management.

In this scenario, augmentation, the regular mixing of individuals between currently isolated populations is the best way to negate the effects of bottlenecks and small population sizes on the genetic diversity of *P. x. xanthopus* (see [4]). Augmentation has been used widely in threatened species management to increase population size and alleviate reduced genetic variation and inbreeding depression and increase reproductive fitness (e.g., [117,118,119]). We recommend the regular augmentation of populations with unrelated wild-caught individuals to improve diversity of currently isolated populations and avoid the ongoing stochastic loss of genetic diversity as opposed to captive breeding, translocations, or reintroductions.

Although our sampling and analysis has demonstrated a lack of contemporary gene flow between most sampled populations, recent (post 2012) evidence of further population growth and expansion in the Gawler and Flinders Ranges (Lethbridge pers. comm.) highlights the need for ongoing genetic sampling, ideally incorporating more widespread population sampling, to enable augmentation to be fine-tuned. If, for example, the recent population growth and dispersal has enabled gene flow between previously isolated populations within range systems to be successfully re-established, then augmentation would only be necessary at a broader scale (e.g., among major clusters of metapopulations). Given the evidence from population viability analysis that some modeled translocations scenarios have no long-term impact on kinship coefficients [43], further genomic sampling and modeling would be useful to inform the best approach for augmentation to have an impact (e.g., numbers, source populations, sex ratios, and required regularity). 

Given our results of recent genetic divergence and the similarity in environments across the semi-arid zone we support moving forward with genetic rescue, with the notion that additional information from these resources be incorporated in the future. Populations are still persisting in situ, therefore, an approach with the least disturbance is the most favorable. Augmentation enhances the adaptive potential of populations and the evolvability of the species as a whole. We note, however, that genetic management needs to be undertaken in combination with effective control of exotic predators [120,121], and thus long-term project management and funding are a necessity. Augmentation needs to consider maintenance of unique diversity within populations (e.g., rare and unique alleles) without swamping populations with new foreign genotypes which have a stronger competitive ability. Given that the environmental conditions across the mountain ranges of the southern arid biome are similar, we propose that the best approach moving forward is to augment populations broadly. We see little likelihood of negative effects of foreign genotypes given the similar environmental conditions, limited historical structure, and the recent divergence of *P. x. xanthopus*. 

In any genetic management (captive breeding, reintroduction, or translocation), the risk of outbreeding depression, where offspring from genetically distant individuals have a lower fitness, needs to be considered. There is strong support for restoring gene flow for small inbred populations isolated by anthropogenic impacts within the last 500 years if the outbreeding risk is low (see [6]). Following the decision-making criterion of [3], we identify that in *P. x. xanthopus* there is a very low risk of outbreeding depression (see Supplementary Figure S5). These populations are recently diverged, do not possess any obvious chromosome differences, the environments are very similar among populations from a climatic perspective, and thus local adaptations should not be extreme. On the basis of previous work, it has been suggested that augmentation not exceed a level of 20% gene flow to reduce losing unique alleles in recipient populations [122]. However, [117] indicate translocation of several individuals per generation should be enough to reduce chances of inbreeding while minimizing risks of outbreeding depression [119,123]. 

According to the available data, we suggest prioritizing augmentation of Gawler Ranges populations with individuals from the Flinders Ranges. The augmentation of some Flinders Ranges populations, particularly Aroona Dam and Sandy Creek which have the lowest diversity, should also be considered. In addition, we propose moving individuals from the Flinders Ranges into Olary Hills to boost the genetic diversity within this population. We suggest initially sourcing individuals from the Flinders Ranges, as this is the more proximal population and reflects the greater connectivity detected from mitochondrial data and genetic differentiation results. On the basis of current data, individuals for augmentation are likely best sourced from Wilkawillina North and Wilkawillina South, as they have the highest allelic richness, allelic diversity, and higher H_O_ than most other populations in the Flinders Ranges. However, long term, individuals should be moved broadly from across populations. Detailed modeling of genetic compatibility and adaptive fitness would assist with augmentation planning moving forward.

### 4.4. Moving Forward

In this study we have focused on neutral genetic markers which provide insight into population dynamics, migration, and effects of genetic drift and inbreeding. However, adaptive genetic diversity, the adaptations to particular environments which are under natural selection play an important role in long-term survival of populations (see [124]). Identification and preservation of such adaptive diversity is important to promote future persistence and adaptive processes and resilience in response to changes, including climate fluctuations [125]. This would be the next step in analyzing the genetic diversity of *P. x. xanthopus* and potentially important for addressing concerns of future adaptability in the face of environmental and climatic change. In addition to evaluating genomic regions under selection, predictive climatic modeling would also provide valuable insight into any local environmental differences between populations and assist in interpreting adaptive variation between populations, as well as physiological and behavioral assessments. Such landscape genomic approaches are showing great promise in conservation biology (e.g., [126]). 

Applying spatial population viability analyses using approximate Bayesian computations would also be a useful decision-planning tool for exploring the outcomes of various management scenarios, as they allow for more complex models to be assessed, incorporating adaptation and selection (see [43]). With incorporation of greater sampling and genetic coverage across the genome, this would allow one to explore the role of local adaptation and neutral processes in maintaining genetic diversity in *P. xanthopus*. 

## 5. Conclusions

Here, we examine the genetic effects of recent declines in *P. x. xanthopus* and highlight how contemporary fragmentation has restricted connectivity among populations. Inference over deeper evolutionary timescales indicates greater connectivity among populations, thus, making the contemporary genetically depauperate populations suited to genetic rescue via augmentation. Incorporation of genetic data in conservation decision making is imperative if management aims to maximize the ability of populations to adapt to future threats and environmental changes. The arid biome of Australia is under-explored and improving our understanding of broad genetic structure across the landscape not only aids single species recovery but also would improve future broad-scale landscape management planning.

## Figures and Tables

**Table 1:** Genetic diversity indices for mitochondrial control region sequences for 11 populations of *P. x. xanthopus*, including: number of samples sequenced (# samples), number of haplotypes in each population (# haplotypes), and the haplotype identifiers (haplotypes), haplotype diversity, nucleotide diversity, and the number of polymorphic sites in a population (# polymorphic sites). Average ± standard deviation.

Population	# Samples	# Haplotypes	Haplotypes	Haplotype Diversity	Nucleotide Diversity	# Polymorphic Sites
1. Aroona Dam	31	1	Z	0.000 ± 0.000	0.00000	0
2. Eregunda	84	2	A (80), F (4)	0.092 ± 0.042	0.00158	11
3. Homestead Range	8	1	H	0.000 ± 0.000	0.00000	0
4. Mt Stuart	3	1	H	0.000 ± 0.000	0.00000	0
5. Sandy Creek	23	4	H (19), L (2), M (1), N (1)	0.320 ± 0.121	0.00243	14
6. Wilkawillina North	31	2	Bt (26), Bc (5)	0.280 ± 0.090	0.00087	2
7. Wilkawillina South	92	6	Bc (72), I3 (9), Bt (6), Q (2), I1 (2), I2 (1)	0.376 ± 0.061	0.00493	14
8. Mt Friday	37	2	K (25), J (12)	0.450 ± 0.057	0.00141	2
9. Yandinga	125	1	G	0.000 ± 0.000	0.00000	0
10. Olary Hills	11	3	C (9), D (1), E (1)	0.182 ± 0.144	0.00401	14
11. Middle Gorge	2	2	R, S	1.000 ± 0.500	0.02184	14

**Table 2:** Genetic diversity indices for microsatellite genotypes for 10 populations of *Petrogale xanthopus xanthopus*, including: the average number of samples analyzed in each population (# samples), allelic diversity, unique alleles, % rare alleles, allelic richness, i.e., the allelic diversity accounting for variation in sample size across populations (in this case accounting for N = 8), observed heterozygosity (H_O_), expected heterozygosity (H_E_), and inbreeding coefficient (F_IS_). Note: the F_IS_ values are all not significant.

Population	# Samples	Allelic Diversity	Unique Alleles	% Rare Alleles	Allelic Richness	H_O_	H_E_	F_IS_
1. Aroona Dam	25	2.76 ± 0.25	0.18 ± 0.10	0.6	2.58 ± 0.82	0.52 ± 0.05	0.46 ± 0.05	−0.095
2. Eregunda	65	5.18 ± 0.38	0.35 ± 0.15	15.9	3.86 ± 1.01	0.65 ± 0.03	0.63 ± 0.03	−0.030
3. Homestead Range	8	3.94 ± 0.29	0.18 ± 0.13	0.0	3.94 ± 1.20	0.73 ± 0.05	0.62 ± 0.03	−0.115
4. Mt Stuart	3	2.53 ± 0.24	0.06 ± 0.06	0.0	-	0.65 ± 0.09	0.46 ± 0.06	−0.234
5. Sandy Creek	17	4.59 ± 0.32	0.12 ± 0.08	11.4	3.74 ± 0.93	0.62 ± 0.05	0.56 ± 0.03	−0.070
6. Wilkawillina North	26	5.12 ± 0.47	0.06 ± 0.06	14.0	4.02 ± 1.24	0.69 ± 0.03	0.63 ± 0.03	−0.074
7. Wilkawillina South	75	6.12 ± 0.49	0.18 ± 0.13	19.7	4.34 ± 1.25	0.69 ± 0.03	0.66 ± 0.03	−0.027
8. Mt Friday	21	3.06 ± 0.26	0.41 ± 0.15	3.8	2.77 ± 0.90	0.52 ± 0.06	0.46 ± 0.05	−0.102
9. Yandinga	91	2.71 ± 0.21	0.18 ± 0.13	5.7	2.23 ± 0.56	0.38 ± 0.05	0.37 ± 0.05	−0.038
10. Olary Hills	11	3.41 ± 0.21	0.35 ± 0.15	5.1	3.26 ± 0.82	0.65 ± 0.05	0.56 ± 0.03	−0.110

**Table 3:** Genetic differentiation between populations of *Petrogale xanthopus xanthopus* with *F_ST_* values above the line and *Φ_ST_* values below the line. Significantly different populations are highlighted in bold. Two populations, Mt Stuart and Middle Gorge, were not included due to small sample size (*N* = 2/3).

	Aroona Dam	Eregunda	Homestead Range	Sandy Creek	Wilkawillina North	Wilkawillina South	Mt Friday	Yandinga	Olary Hills
Aroona Dam	-	0.300	0.333	0.365	0.324	0.272	0.390	0.498	0.369
Eregunda	0.959	-	0.182	0.236	0.126	0.101	0.271	0.379	0.196
Homestead Range	1.000	0.798	-	0.160	0.166	0.153	0.311	0.457	0.193
Sandy Creek	0.748	0.748	0.000	-	0.196	0.185	0.306	0.478	0.198
Wilkawillina North	0.980	0.911	0.946	0.865	-	0.050	0.330	0.397	0.175
Wilkawillina South	0.851	0.793	0.693	0.687	0.263	-	0.279	0.341	0.167
Mt Friday	0.973	0.926	0.935	0.886	0.898	0.735	-	0.446	0.280
Yandinga	1.000	0.828	1.000	0.969	0.982	0.828	0.885	-	0.442
Olary Hills	0.955	0.926	0.885	0.848	0.916	0.779	0.919	0.986	-

## Supplementary Materials

The following are available online at https://www.mdpi.com/2073-4425/11/2/154/s1, Supplementary Figure S1: Principal coordinate analysis of populations of P. x. xanthopus based on microsatellite genotypes. Supplementary Figure S2: Mean pairwise relatedness for individual male (above) and female (below) P. x. xanthopus from populations in South Australia. Supplementary Figure S3: Phylogenetic tree based on maximum likelihood analysis of mitochondrial control region haplotypes of P. x. xanthopus (yellow) and P. x. celeris (red). Supplementary Figure S4: Mantel tests for isolation-by-distance of log geographic distance against genetic distance. Supplementary Figure S5: Decision framework based on genetic data. Supplementary Table S1: Sample Information. Supplementary Table S2: Mitochondrial control region sequence divergence. Supplementary Table S3: Results for statistical analysis of neutrality. Supplementary Table S4: Bottleneck results. Supplementary Table S5: First generation migrants. Supplementary Table S6: Evidence of admixture and gene flow amongst populations of *P. x. xanthopus*.
