# Supplementary material for: Understanding Historical Demographic Processes to Inform Contemporary Conservation of an Arid Zone Specialist: The Yellow-Footed Rock-Wallaby
Source: Genes (Basel). 2020 Jan 31;11(2):154. doi: 10.3390/genes11020154 (PMC7073556; doi:10.3390/genes11020154)
Supplement: Supplementary file 1 [file genes-11-00154-s001.zip › Supplementary Files/SuppTable5_First generation migrants.docx]

**Table 4** First generation migrants detected from GeneClass2 analysis. Results include the population samples (ID), where they were sourced from, their sex, the log value of the ratio of the likelihood of the individual within the population it is sampled (L_home) to the highest likelihood value among all sampled populations including the population where the individual was sampled (L_max), the probability of the log value, the log likelihood of the population the individual is sampled (-log(L) home), the log of the highest likelihood of populations for the analysis and the associated population of this highest likelihood (population immigrated from).

| **Population Sourced** | **ID** | **Sex** | **Log(L_home / L_max)** | **probability** | **-log(L) home** | **-log(L) best** | **Population immigrated from** |
| --- | --- | --- | --- | --- | --- | --- | --- |
| Sandy Creek | 2304 | male | 3.728 | 0.006 | 30.393 | 26.665 | Homestead Range |
| Sandy Creek | 2307 | male | 14.736 | 0 | 38.864 | 24.128 | Wilkawillina North |
| Sandy Creek | 2309 | male | 4.713 | 0.006 | 34.779 | 30.067 | Wilkawillina South |
| Wilkawillina North | 1675 | male | 0.706 | 0.062 | 16.057 | 15.351 | Wilkawillina South |
| Wilkawillina North | 1678 | male | 4.773 | 0.005 | 19.863 | 15.089 | Wilkawillina South |
| Wilkawillina North | 1680 | male | 1.045 | 0.055 | 18.389 | 17.344 | Wilkawillina South |
| Wilkawillina North | 1931 | female | 1.431 | 0.039 | 17.657 | 16.227 | Wilkawillina South |
| Wilkawillina North | 2315 | male | 7.455 | 0 | 23.554 | 16.099 | Wilkawillina South |
| Wilkawillina South | 1733 | male | 0.46 | 0.03 | 17.663 | 17.203 | Wilkawillina North |
| Wilkawillina South | 2335 | unknown | 1.134 | 0.019 | 23.479 | 22.345 | Wilkawillina North |
| Wilkawillina South | 2339 | unknown | 1.82 | 0.011 | 17.689 | 15.869 | Wilkawillina North |
| Wilkawillina South | 2347 | unknown | 3.498 | 0.002 | 17.675 | 14.177 | Wilkawillina North |
| Wilkawillina South | 2352 | male | 2.887 | 0.005 | 16.33 | 13.444 | Wilkawillina North |
