# Supplementary material for: Understanding Historical Demographic Processes to Inform Contemporary Conservation of an Arid Zone Specialist: The Yellow-Footed Rock-Wallaby
Source: Genes (Basel). 2020 Jan 31;11(2):154. doi: 10.3390/genes11020154 (PMC7073556; doi:10.3390/genes11020154)
Supplement: Supplementary file 1 [file genes-11-00154-s001.zip › Supplementary Files/SuppTable6_Evidence of admixture and gene flow amongst populations of Petrogale xanthopus xanthopus.docx]

**Supplementary Table 4** Evidence of admixture and gene flow amongst populations of *Petrogale xanthopus xanthopus*. (a) Evidence of admixture from Structure analysis included the highest probability for belonging to a population other than that which the individual was sampled with a minimum probability of 0.5. The alternate population from which the genetic data supports clustering is listed along with the sex and whether there is also support from GeneClass2 for evidence of being a first generation migrant. (b) Information in relation to the mtDNA haplotype of admixed/first generation migrant individuals and the most common mtDNA haplotype from the population which they cluster together with genetically, aside from the population they were trapped. In addition, individual’s age when they were trapped, the years trapped and additional comments.

(a)

| **Sample ID** | **Population trapped** | **Sex** | **Structure evidence of admixture** | **GeneClass first generation migrant** | **Highest Probability for Structure population** | **Population admixed from Structure** |
| --- | --- | --- | --- | --- | --- | --- |
| 1673 | Wilkawillina North | female | ✓ | ✗ | 0.8717 | Wilkawillina South |
| 1675 | Wilkawillina North | male | ✓ | ✓ | 0.9672 | Wilkawillina South |
| 1678 | Wilkawillina North | male | ✓ | ✓ | 0.9673 | Wilkawillina South |
| 1680 | Wilkawillina North | male | ✓ | ✓ | 0.9579 | Wilkawillina South |
| 1706 | Wilkawillina North | male | ✓ | ✗ | 0.5369 | Wilkawillina South |
| 1931 | Wilkawillina North | female | ✓ | ✓ | 0.8489 | Wilkawillina South |
| 2315 | Wilkawillina North | male | ✓ | ✓ | 0.979 | Wilkawillina South |
| 1733 | Wilkawillina South | male | ✗ | ✓ | 0.8648 | Wilkawillina South |
| 2335 | Wilkawillina South | unknown | ✓ | ✓ | 0.8627 | Wilkawillina North |
| 2339 | Wilkawillina South | unknown | ✗ | ✓ | 0.6001 | Wilkawillina South |
| 2342 | Wilkawillina South | male | ✓ | ✗ | 0.6085 | Wilkawillina North |
| 2344 | Wilkawillina South | male | ✓ | ✗ | 0.5722 | Wilkawillina North |
| 2346 | Wilkawillina South | unknown | ✓ | ✗ | 0.8664 | Wilkawillina North |
| 2347 | Wilkawillina South | unknown | ✓ | ✓ | 0.9467 | Wilkawillina North |
| 2352 | Wilkawillina South | male | ✓ | ✓ | 0.8381 | Wilkawillina North |
| 2304 | Sandy Creek | male | ✓ | ✓ | 0.6291 | Homestead Range |
| 2307 | Sandy Creek | male | ✓ | ✓ | 0.438 | Eregunda |
| 2309 | Sandy Creek | male | ✓ | ✓ | 0.3552 | Wilkawillina South |
| 1885 | Mt Stuart | unknown | ✓ | ✗ | 0.6274 | Wilkawillina South |

| **Sample ID** | **Population trapped** | **mtDNA haplotype** | **Most common mtDNA haplotype of source population** | **# Rare alleles** | **Age of animal at first capture** | **Date/s trapped** | **Comments** |
| --- | --- | --- | --- | --- | --- | --- | --- |
| 1673 | Wilkawillina North | Bt | Bc (Wilkawillina South) | 1 (3.2%) | subadult | 1999 | * twice in 1999 |
| 1675 | Wilkawillina North | Bc | Bc (Wilkawillina South) | 2 (6.5%) | adult | 1999-2002 |  |
| 1678 | Wilkawillina North | Bt | Bc (Wilkawillina South) | 1 (3.2%) | adult | 1999-2000 |  |
| 1680 | Wilkawillina North | Bc | Bc (Wilkawillina South) | 3 (9.7%) | adult | 1999-2001 |  |
| 1706 | Wilkawillina North | Bc | Bc (Wilkawillina South) | 2 (6.5%) | adult | 1999-2002 |  |
| 1931 | Wilkawillina North | Bt | Bc (Wilkawillina South) | 0 (0.0%) | adult | 2004 | * single appearance |
| 2315 | Wilkawillina North | Bc | Bc (Wilkawillina South) | 1 (3.2%) | adult | 2005, 2008 |  |
| 1733 | Wilkawillina South | Bc | Bc (Wilkawillina South) | 3 (13.6%) | adult | 2000 | * single appearance |
| 2335 | Wilkawillina South | I2 | Bt (Wilkawillina North) | 3 (13.6%) | ? | ? |  |
| 2339 | Wilkawillina South | Bt | Bc (Wilkawillina South) | 1 (4.5%) | ? | ? |  |
| 2342 | Wilkawillina South | Bc | Bt (Wilkawillina North) | 4 (18.2%) | subadult | 2007 | * single appearance |
| 2344 | Wilkawillina South | Bc | Bt (Wilkawillina North) | 1 (4.5%) | adult | 2007-2009 |  |
| 2346 | Wilkawillina South | Bc | Bt (Wilkawillina North) | 1 (4.5%) | ? | ? |  |
| 2347 | Wilkawillina South | Bt | Bt (Wilkawillina North) | 0 (0.0%) | ? | ? |  |
| 2352 | Wilkawillina South | Bc | Bt (Wilkawillina North) | 0 (0.0%) | adult | 2009-2010 |  |
| 2304 | Sandy Creek | L | H (Homestead Range) | - | adult | 2010 |  |
| 2307 | Sandy Creek | M | A (Eregunda) | 1 (4.0%) | adult | 2010 |  |
| 2309 | Sandy Creek | N | Bc (Wilkawillina South) | 4 (12.9%) | adult | 2010 |  |
| 1885 | Mt Stuart | H | Bc (Wilkawillina South) | 3 (9.7%) | ? | ? |  |

(b)
