# Supplementary material for: Understanding Historical Demographic Processes to Inform Contemporary Conservation of an Arid Zone Specialist: The Yellow-Footed Rock-Wallaby
Source: Genes (Basel). 2020 Jan 31;11(2):154. doi: 10.3390/genes11020154 (PMC7073556; doi:10.3390/genes11020154)

Supplementary Figure 1a Principal coordinate analysis of populations of P. x. xanthopus based on microsatellite genotypes.

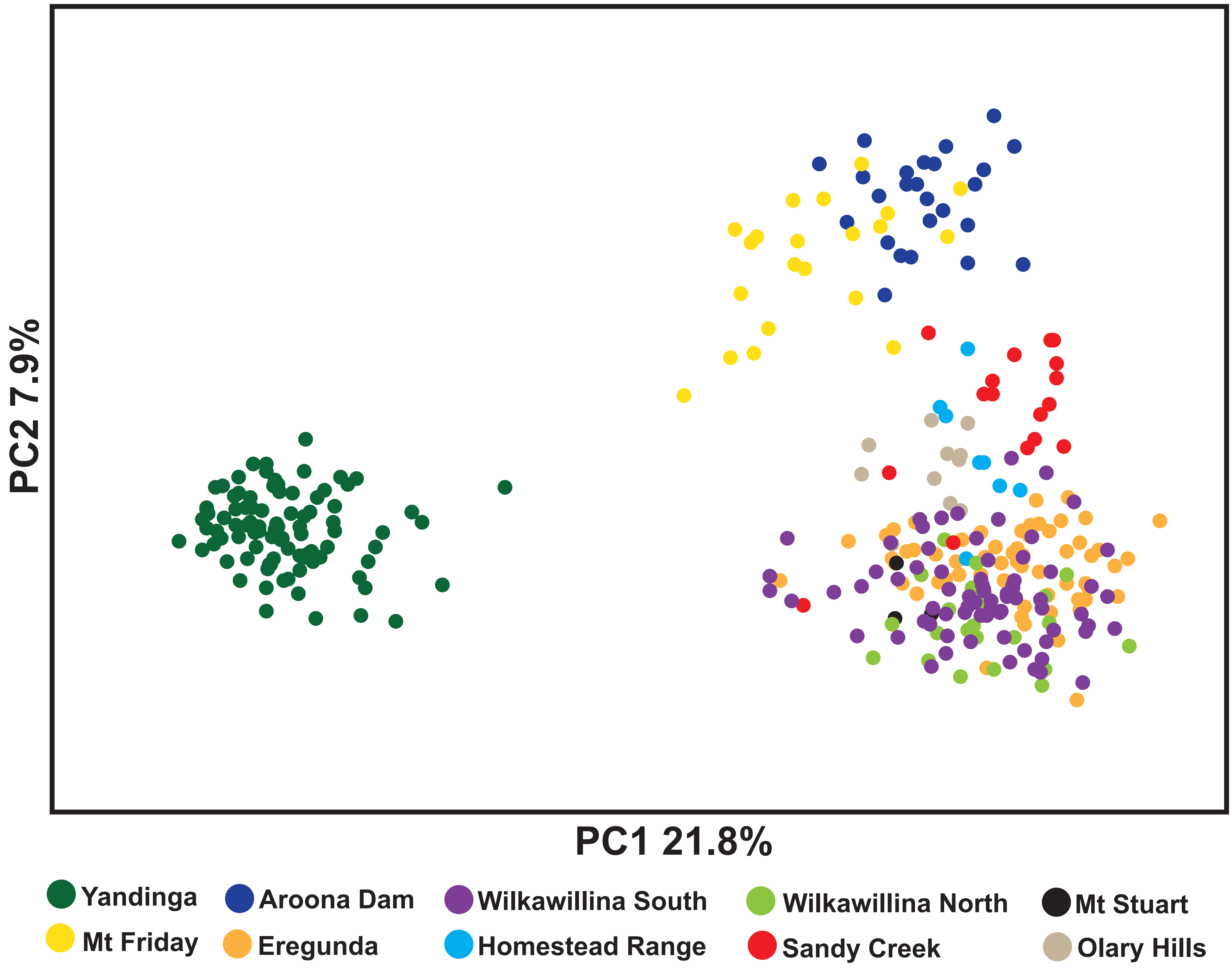

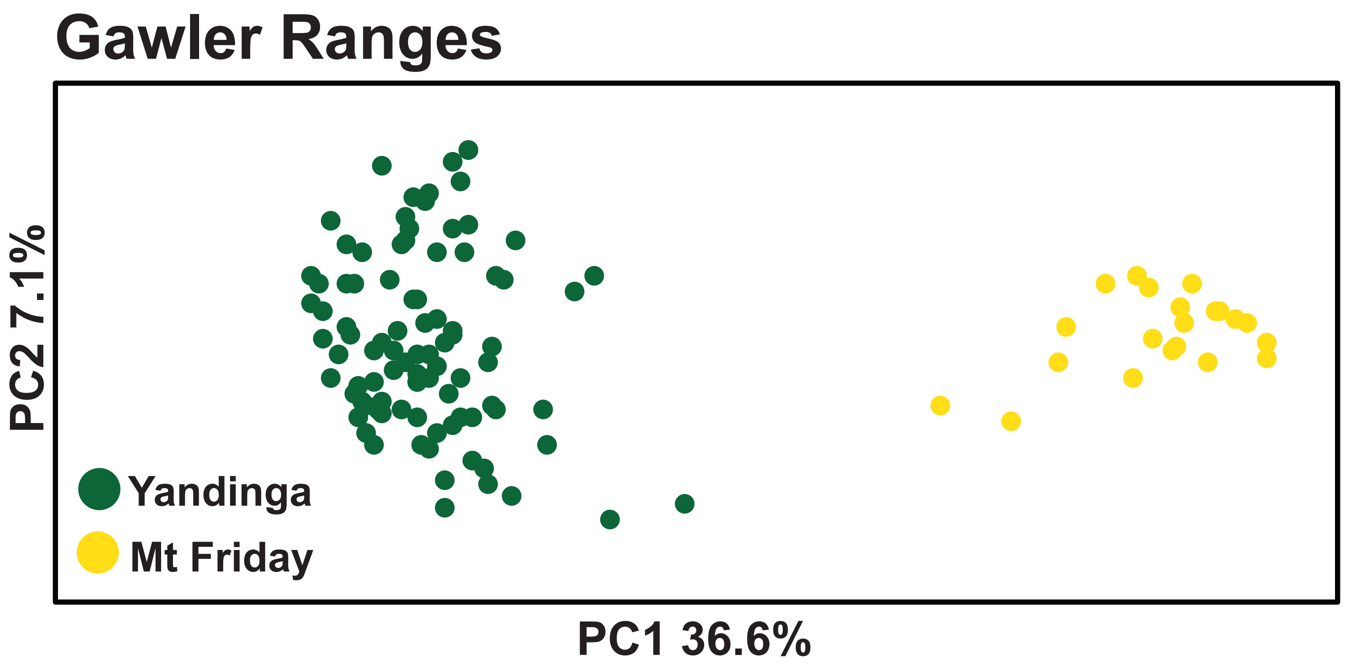

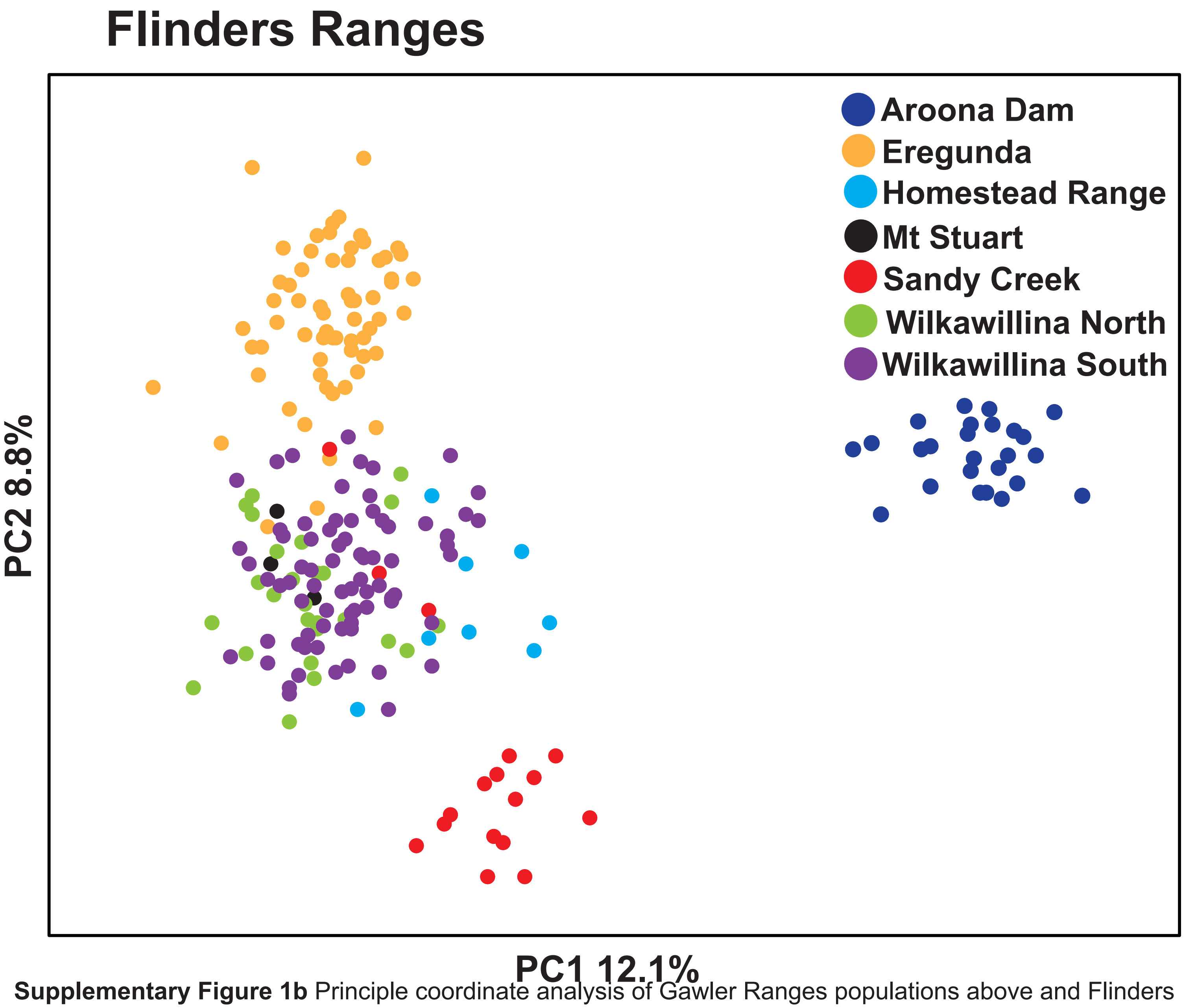

Supplementary Figure 1b Principle coordinate analysis of Gawler Ranges populations above and Flinders Ranges populations of P. x. xanthopus below based on microsatellite genotypes.

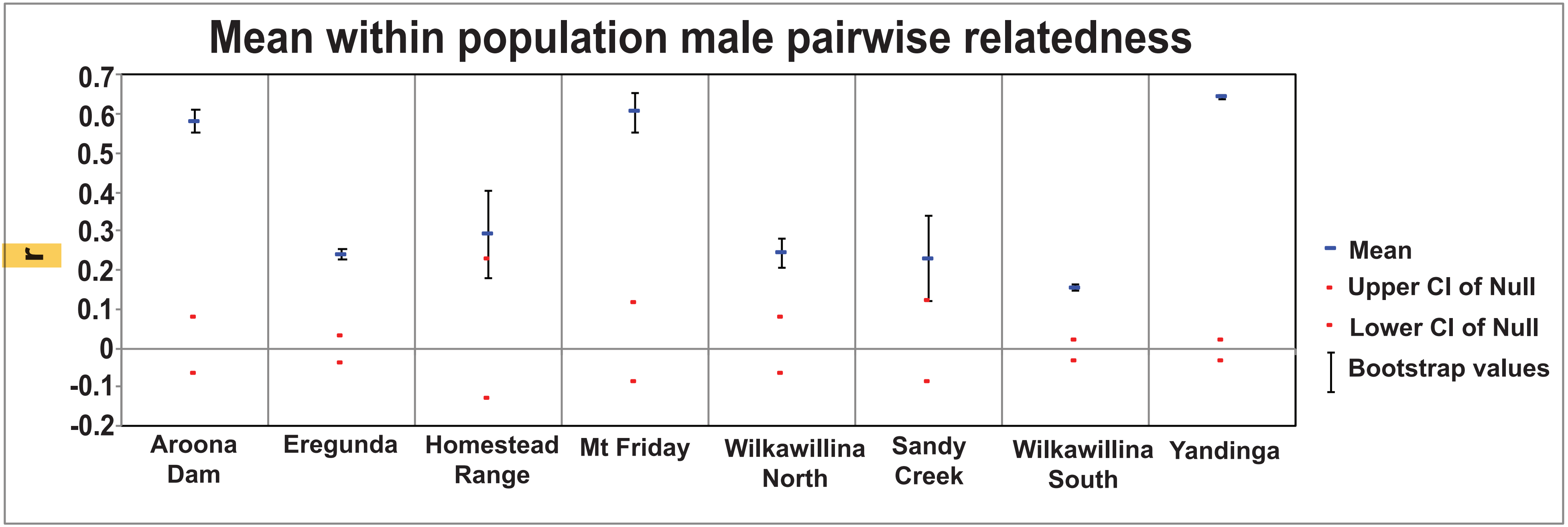

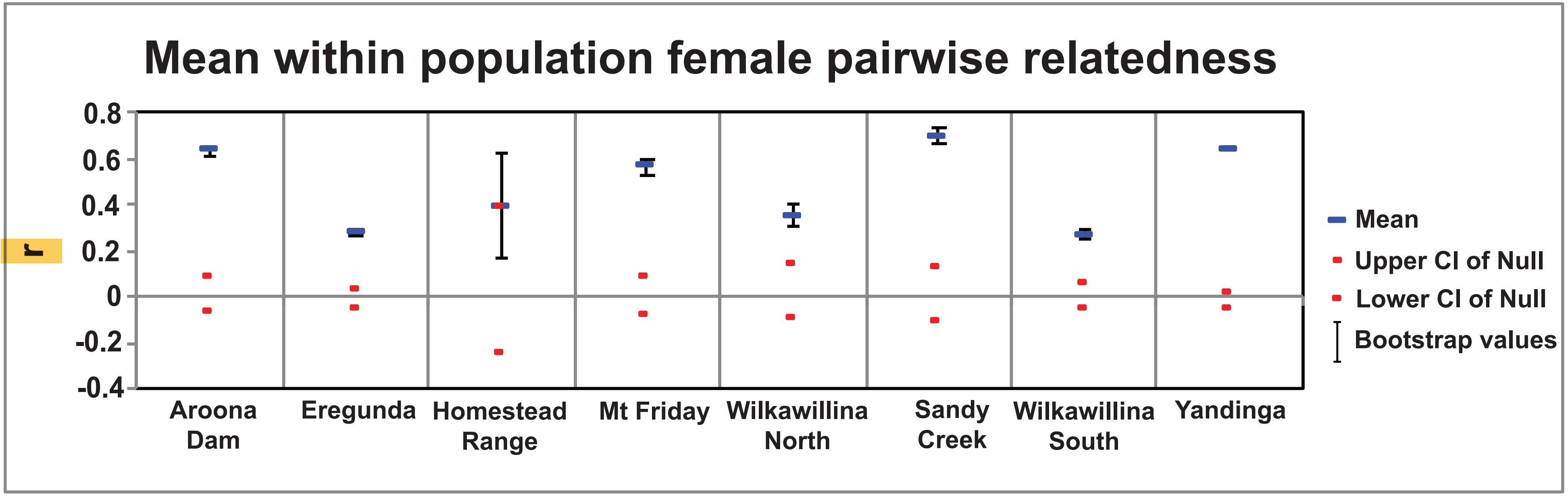

Supplementary Figure 2 Mean pairwise relatedness for individual male (above) and female (below) P. x. xanthopus from populations in South Australia. Error bars surround the mean pairwise relatedness value (blue line) based on bootstrap analysis; and red lines represent the 95% upper and lower confidence bounds around the null hypothesis of no difference across populations.

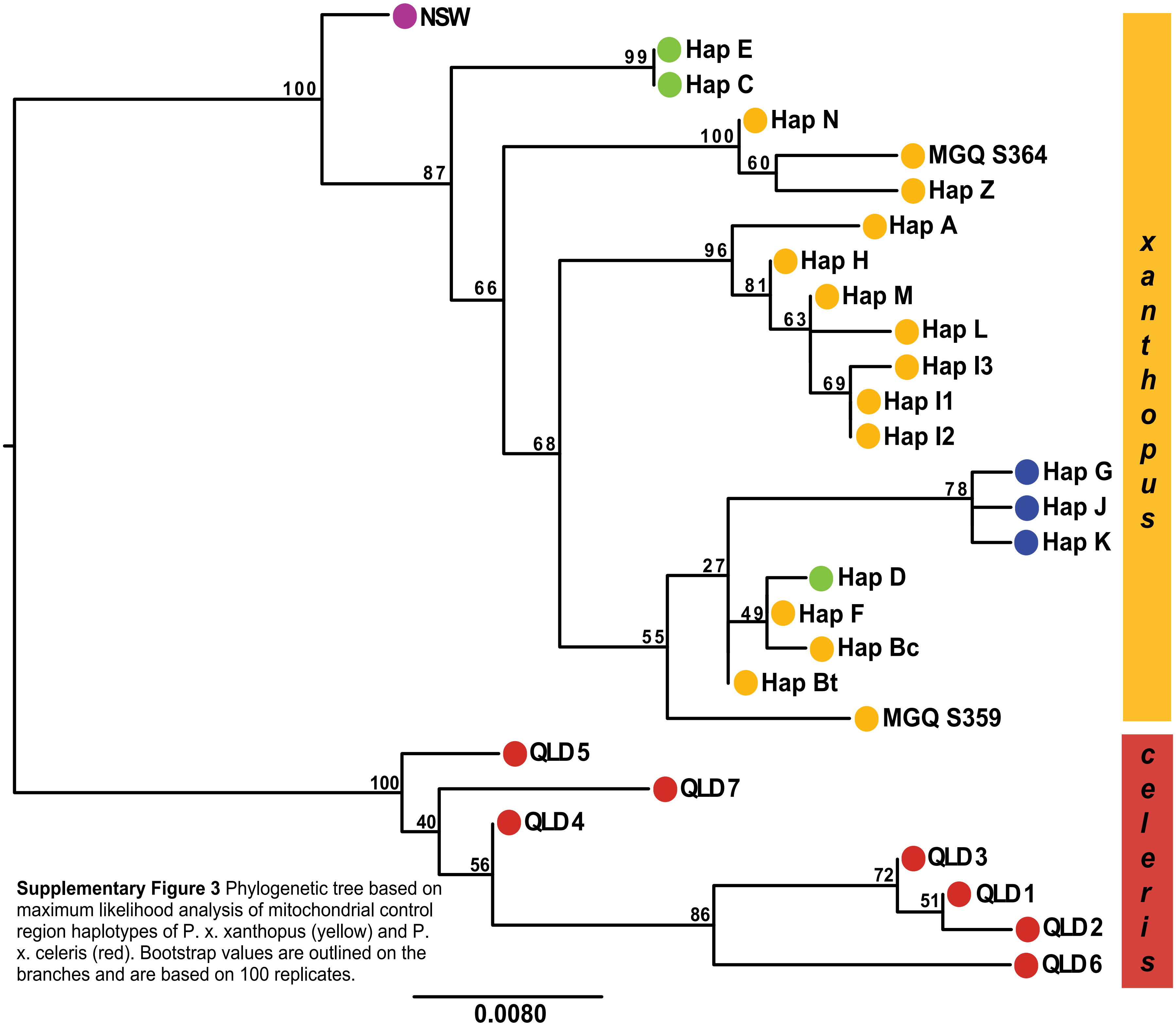

Supplementary Figure 3 Phylogenetic tree based on maximum likelihood analysis of mitochondrial control region haplotypes of P. x. xanthopus (yellow) and P. x. celeris (red). Bootstrap values are outlined on the branches and are based on 100 replicates.

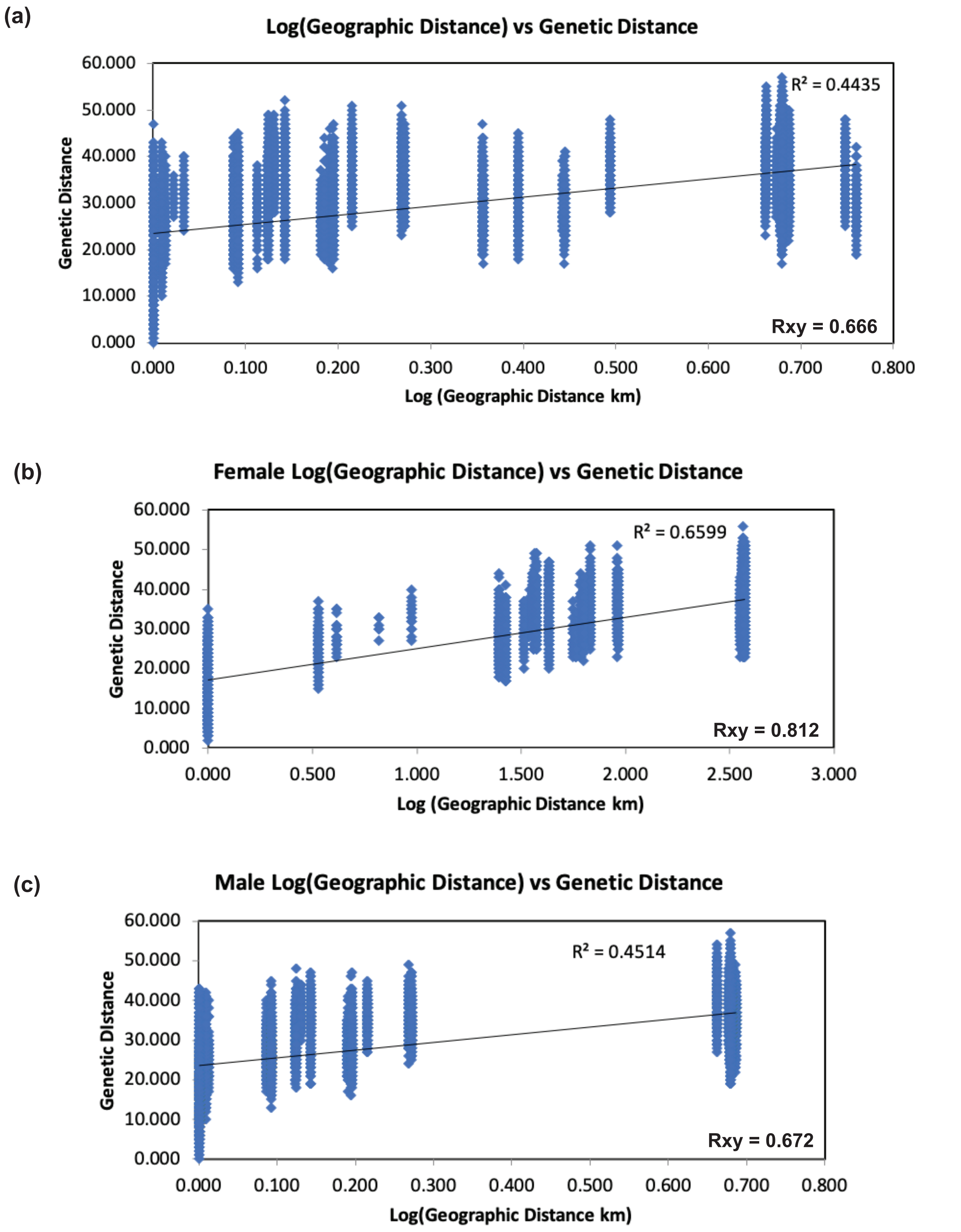

Supplementary Figure 4 Mantel tests for isolation-by-distance of log geographic distance against genetic distance for (a) all indiviudals, (b) males only, and (c)females only. All were significantly correlated.

Supplementary Figure 5 Decision framework from Frankham et al. (2011) highlighting the evaluation process for management based on genetic data.

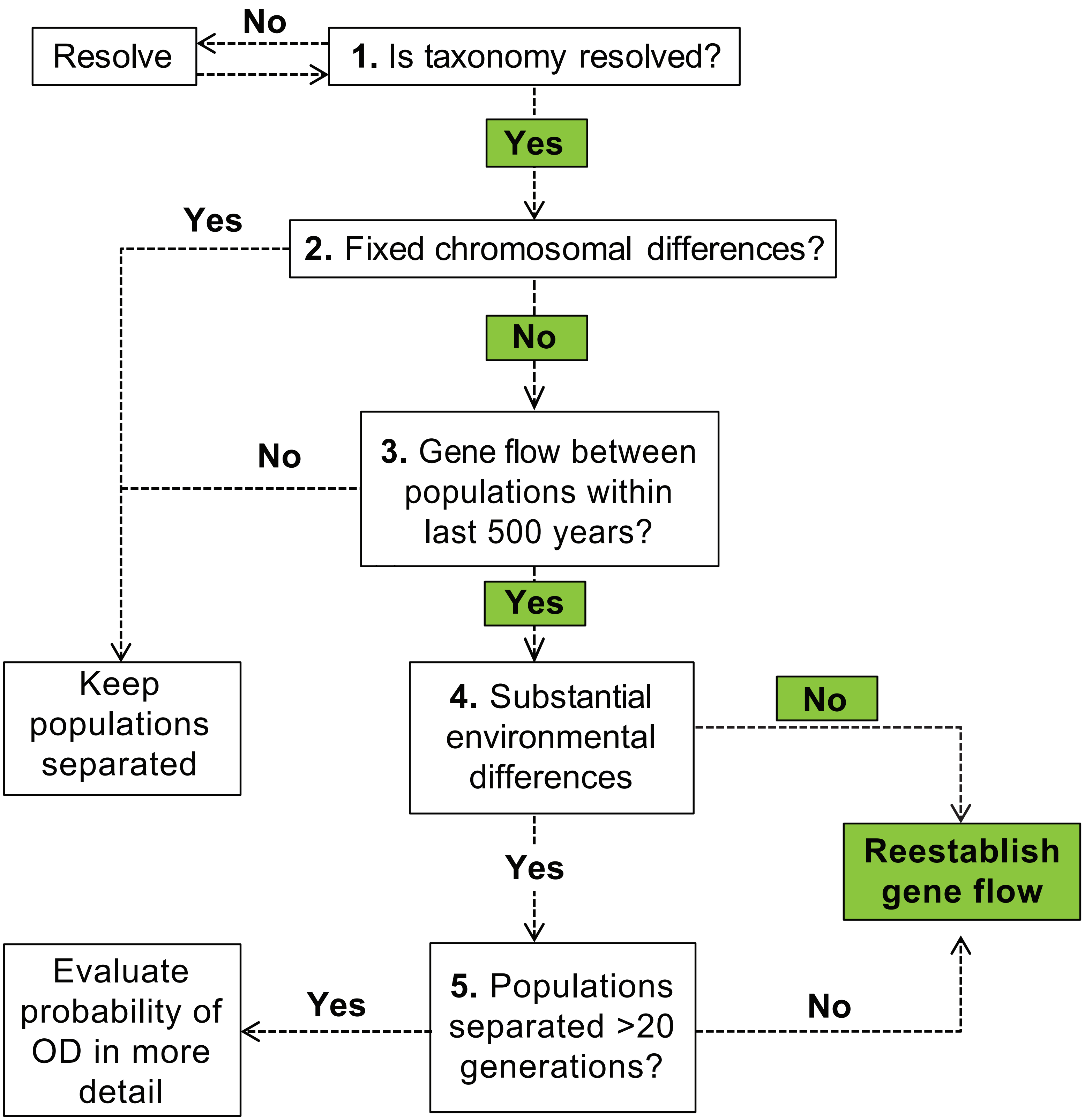

Supplement: Supplementary file 1 [file genes-11-00154-s001.zip › Supplementary Files/Combined_Supp_Figs_UPDATED.pdf]
