# Supplementary material for: Understanding Historical Demographic Processes to Inform Contemporary Conservation of an Arid Zone Specialist: The Yellow-Footed Rock-Wallaby
Source: Genes (Basel). 2020 Jan 31;11(2):154. doi: 10.3390/genes11020154 (PMC7073556; doi:10.3390/genes11020154)
Supplement: Supplementary file 1 [file genes-11-00154-s001.zip › Supplementary Files/SuppTable2_Mitochondrial control region sequence divergence.docx]

**Supplementary Table 2** Mitochondrial control region sequence divergence (D*xy*) amongst populations of *Petrogale xanthopus xanthopus*.

|  | **Aroona Dam** | **Eregunda** | **Homestead Range** | **Mt Stuart** | **Sandy Creek** | **Wilkawillina North** | **Wilkawillina South** | **Mt Friday** | **Yandinga** | **Olary Hills** | **MG** |
| --- | --- | --- | --- | --- | --- | --- | --- | --- | --- | --- | --- |
| **Aroona Dam** |  |  |  |  |  |  |  |  |  |  |  |
| **Eregunda** | 0.02478 |  |  |  |  |  |  |  |  |  |  |
| **Homestead Range** | 0.02188 | 0.00662 |  |  |  |  |  |  |  |  |  |
| **Mt Stuart** | 0.02188 | 0.00662 | 0.00000 |  |  |  |  |  |  |  |  |
| **Sandy Creek** | 0.02053 | 0.00637 | 0.00124 | 0.00124 |  |  |  |  |  |  |  |
| **Wilkawillina North** | 0.01925 | 0.01544 | 0.01300 | 0.01300 | 0.01233 |  |  |  |  |  |  |
| **Wilkawillina South** | 0.02230 | 0.01688 | 0.01400 | 0.01400 | 0.01357 | 0.00444 |  |  |  |  |  |
| **Mt Friday** | 0.02555 | 0.02046 | 0.01774 | 0.01774 | 0.01694 | 0.01144 | 0.02046 |  |  |  |  |
| **Yandinga** | 0.02500 | 0.01838 | 0.01563 | 0.01563 | 0.01486 | 0.00988 | 0.01838 | 0.00313 |  |  |  |
| **Olary Hills** | 0.02491 | 0.02165 | 0.01864 | 0.01864 | 0.01794 | 0.01749 | 0.01827 | 0.02187 | 0.02122 |  |  |
| **Middle Gorge** | 0.01560 | 0.02463 | 0.02188 | 0.02188 | 0.02074 | 0.01588 | 0.01832 | 0.02449 | 0.02344 | 0.02176 |  |
| **Outgroups** | 0.16388 | 0.15602 | 0.15410 | 0.15410 | 0.15559 | 0.16107 | 0.16133 | 0.16250 | 0.16164 | 0.16172 | 0.16221 |
