# Supplementary material for: Understanding Historical Demographic Processes to Inform Contemporary Conservation of an Arid Zone Specialist: The Yellow-Footed Rock-Wallaby
Source: Genes (Basel). 2020 Jan 31;11(2):154. doi: 10.3390/genes11020154 (PMC7073556; doi:10.3390/genes11020154)
Supplement: Supplementary file 1 [file genes-11-00154-s001.zip › Supplementary Files/SuppTable3_Table of results for statistical analysis of neutrality.docx]

**Supplementary Table 3** Table of results for statistical analysis of non-neutral processes of evolution, including Tajima’s D, Fu’s Fs and R2. These analyses were run on the mitochondrial control region dataset for each population. Statistics could not be computed for Aroona Dam, Homestead Range and Yandinga due to no polymorphisms in the dataset. In addition, Mt Stuart could not be included due to too low a sample size. There is evidence of selection or non-neutral processes based on Tajima’s D for Sandy Creek and Olary Hills. There is significant evidence of population expansion from Fu’s Fs for Wilkawillina South and Olary Hills. Coalescent simulations indicate support for a lower Tajima’s D value than that estimated for Eregunda, Sandy Creek and Olary Hills. Coalescent simulations do not support a lower Fu’s Fs or R2 value than that estimated.

| **Population** | **Tajima's D** | **Significance** | **Simulations** | **Fu's Fs** | **Significance** | **Simulations** | **R2** | **Simulations** |
| --- | --- | --- | --- | --- | --- | --- | --- | --- |
| Eregunda | -1.44729 | NS P > 0.10 | P [D <= -1.4473]: 0.00000 | 4.458 | NS 0.058 | P [Fs <= 4.4584]: 0.94800 | 0.0459 | P [R2 <= 0.0459]: 0.07746 |
| Sandy Creek | -2.11498 | S P <0.05 | P [D <= -2.1150]: 0.00500 | 1.211 | NS 0.23 | P [Fs <= 1.2109]: 0.74400 | 0.1615 | P [R2 <= 0.1615]: 0.73860 |
| Wilkawillina North | 0.23707 | NS P > 0.10 | P [D <= 0.2371]: 0.64600 | 1.893 | NS 0.292 | P [Fs <= 1.8928]: 0.90900 | 0.1398 | P [R2 <= 0.1398]: 0.52571 |
| Wilkawillina South | 0.40512 | NS P > 0.10 | P [D <= 0.4051]: 0.72200 | 5.733 | S 0.009 | P [Fs <= 5.7333]: 0.96300 | 0.1125 | P [R2 <= 0.1125]: 0.73800 |
| Mt Friday | 1.69783 | NS P > 0.10 | P [D <= 1.6978]: 0.94900 | 3.253 | NS 0.14 | P [Fs <= 3.2526]: 0.97000 | 0.2252 | P [R2 <= 0.2252]: 0.94100 |
| Olary Hills | -2.06008 | S P <0.01 | P [D <= -2.0601]: 0.00300 | 5.014 | S 0.049 | P [Fs <= 5.0143]: 0.98900 | 0.2875 | P [R2 <= 0.2875]: 1.00000 |
