# Supplementary material for: Understanding Historical Demographic Processes to Inform Contemporary Conservation of an Arid Zone Specialist: The Yellow-Footed Rock-Wallaby
Source: Genes (Basel). 2020 Jan 31;11(2):154. doi: 10.3390/genes11020154 (PMC7073556; doi:10.3390/genes11020154)
Supplement: Supplementary file 1 [file genes-11-00154-s001.zip › Supplementary Files/SuppTable4_Bottleneck results.docx]

**Supplementary Table 4** Bottleneck results : N = sample size, *k* = number of alleles, He = heterozygosities found in iterations producing exactly *k* alleles, sign two phase model (TPM).

|  | Mean N | Mean *k* | Mean He | Sign TPM | Sign TPM |
| --- | --- | --- | --- | --- | --- |
| Aroona Dam | 50 | 2.76 | 0.473 | 0.019* | 0.002* |
| Eregunda | 129.88 | 5.18 | 0.635 | 0.098 | 0.006* |
| Homestead Range | 16 | 3.94 | 0.658 | 0.028* | 0.008* |
| Mt Stuart | 6 | 2.53 | 0.549 | 0.473 | 0.370 |
| Sandy Creek | 33.88 | 4.59 | 0.578 | 0.413 | 0.365 |
| Wilkawillina North | 51.88 | 5.12 | 0.645 | 0.427 | 0.029* |
| Wilkawillina South | 148.71 | 6.12 | 0.669 | 0.032* | 0.007* |
| Mt Friday | 41.88 | 3.06 | 0.475 | 0.032* | 0.019* |
| Yandinga | 181.76 | 2.71 | 0.370 | 0.061 | 0.010* |
| Olary Hills | 22 | 3.41 | 0.591 | 0.092 | 0.061 |
